# Identifying Frameworks for Validation and Monitoring of Consensual Behavioral Intervention Technologies: Narrative Review

**DOI:** 10.2196/13606

**Published:** 2019-10-16

**Authors:** François Carbonnel, Gregory Ninot

**Affiliations:** 1 Research Unit EA4556 Epsylon University of Montpellier Montpellier France; 2 Plateforme Universitaire Collaborative d’Evaluation des Programmes de Prévention et de Soins de Support University of Montpellier Montpellier France; 3 University Department of General Practice University of Montpellier Montpellier France; 4 University Multiprofessional Health Center Avicenne Perpignan France; 5 Montpellier Cancer Institute Montpellier France

**Keywords:** behavioral intervention technology, validation, surveillance, paradigm, framework, nonpharmacological interventions

## Abstract

**Background:**

Changing health behaviors, such as smoking, unhealthy eating, inactivity, and alcohol abuse, may have a greater impact on population health than any curative strategy. One of the suggested strategies is the use of behavioral intervention technologies (BITs). They open up new opportunities in the area of prevention and therapy and have begun to show benefits in the durable change of health behaviors in patients or those at risk. A consensual and international paradigm was adopted by health authorities for drugs 50 years ago. It guides their development from research units to their authorization and surveillance. BITs’ generalization brings into question their upstream evaluation before being placed on the market and their downstream monitoring once on the market; this is especially the case in view of the marketing information provided by manufacturers and the scarcity and methodological limits of scientific studies on these tools.

**Objective:**

This study aims to identify and categorize the frameworks for the validation and monitoring of BITs proposed in the literature.

**Methods:**

We conducted a narrative literature review using MEDLINE, PsycINFO, and Web of Science. The review items included the following: name, publication year, name of the creator (ie, first author), country, funding organization, health focus, target group, and design (ie, linear, iterative, evolutive, and/or concurrent). The frameworks were then categorized based on (1) translational research thanks to a continuum of steps and (2) the three paradigms that may have inspired the frameworks: biomedical, engineering, and/or behavioral.

**Results:**

We identified 46 frameworks besides the classic US Food and Drug Administration (FDA) five-phase drug development model. A total of 57% (26/46) of frameworks were created in the 2010s and 61% (28/46) involved the final user in an early and systematic way. A total of 4% (2/46) of frameworks had a linear-only sequence of their phases, 37% (17/46) had a linear and iterative structure, 33% (15/46) added an evolutive structure, and 24% (11/46) were associated with a parallel process. Only 12 out of 46 (26%) frameworks covered the continuum of steps and 12 (26%) relied on the three paradigms.

**Conclusions:**

To date, 46 frameworks of BIT validation and surveillance coexist, besides the classic FDA five-phase drug development model, without the predominance of one of them or convergence in a consensual model. Their number has increased exponentially in the last three decades. Three dangerous scenarios are possible: (1) anarchic continuous development of BITs that depend on companies amalgamating health benefits and usability (ie, user experience, data security, and ergonomics) and limiting implementation to several countries; (2) the movement toward the type of framework for drug evaluation centered on establishing its effectiveness before marketing authorization to guarantee its safety for users, which is heavy and costly; and (3) the implementation of a framework reliant on big data analysis based on a posteriori research and an autoregulation of a market, but that does not address the safety risk for the health user, as the market will not regulate safety or efficacy issues. This paper recommends convergence toward an international validation and surveillance framework based on the specificities of BITs, not equivalent to medical devices, to guarantee their effectiveness and safety for users.

## Introduction

According to the World Health Organization (WHO), changing health-related behaviors, such as smoking, unhealthy diet, physical inactivity, and alcohol abuse, could avoid up to 80% of heart diseases, strokes, and type 2 diabetes as well as more than 30% of cancers [1]. These behaviors explain 50% of premature mortality and morbidity in the United States [2]. Improving the efficacy of the interventions dedicated to sustainably change these behaviors will have a greater impact on population health than any therapeutic strategy [3]. One of the possible solutions is the use of nonpharmacological interventions such as digital health interventions [4]. Among eHealth tools, mHealth includes mobile phone health apps and connected health devices [5,6]. Between 20% and 80% of adults are equipped with connected health devices, from one country to another [7-11]. Behavioral intervention technologies (BITs) “employ a broad range of technologies, such as mobile phones, the Web, and sensors, to support users in changing behaviors and cognitions related to health, mental health, and wellness” [12]. Digital health interventions open up new opportunities in the area of prevention and therapeutics: quantified-self activities and behaviors; sharing of clinical, psychological, and behavioral data (ie, short message service [SMS] and social networks); real-time analysis of health data; delivering of health promotion messages (ie, mobile phone and Web apps); involvement of health professionals (ie, telemedicine); e-coaching; social support of the family; and peers in the social environment [13,14]. Several observational studies indicate benefits of BITs on health behavior change in patients with chronic disease or at-risk people using interventions to assist with stronger drug compliance, smoking cessation, alcohol consumption reduction, weight management, and better diets [15-18]. Does the existence of a benefit provide evidence of an effective and safe tool and the only approach to generalize it?

The evaluation of BITs before market and their ongoing monitoring remain questionable, especially in a world propelled by marketing strategies and the lack of regulation of health solutions that do not belong to the category of medical devices. According to the US Food and Drug Administration (FDA) [19,20], the validation process for drugs and implantable medical devices involves the “collection and evaluation of data, from the process design stage through commercial production, which establishes scientific evidence that a process is capable of consistently delivering quality product.” To assess the efficacy and safety of a health product, there is a need to use a specific scientific paradigm, which is “a set of principles and methods shared by a scientific community” [21]. A framework is a model for planning processes or for action plans, which brings a systematic approach to developing, managing, and evaluating interventions [22]. Regulators, researchers, and manufacturers share a consensual paradigm for drugs [19,20]. This *clinical trial* framework guides the development from lab to authorization and monitoring. It is organized in five phases: Phase 0 (ie, preclinical) to identify mechanisms; Phase 1 to determine tolerance in healthy humans; Phase 2 to identify the optimal dose for a small number of patients (ie, pilot trial); Phase 3 to demonstrate evidence of efficacy and safety (ie, randomized controlled trial); and Phase 4 to ensure long-term safety [23]. The number of studies assessing BITs is growing exponentially. However, their methodological designs do not follow an established validation framework. They flow from individual or research team choice, context, and/or opportunity.

The objective of this study was to identify the frameworks for the validation and monitoring of BITs proposed in the literature, besides the five-phase drug development model, and to categorize them.

## Methods

### Data Collection

We have conducted a narrative literature review of articles published up to April 4, 2019, of the validation and monitoring frameworks of BITs. The main databases that we searched were MEDLINE, PsycINFO, Web of Science, Science Direct, and the Journal of Medical Internet Research (JMIR) database. They have been chosen as reference databases in biomedicine and psychology and comprise the largest general scientific database [24]. The search keywords were as follows: (“model” OR “framework” OR “process” OR “evaluation process” OR “validation process”) AND (“behavior” OR “behavior change” OR “behavioral intervention”) AND (“digital health” OR “ehealth” OR “mhealth” OR “connected health” OR “medical app” OR “smartphone” OR “iphone” OR “email” OR “text message” OR “SMS” OR “mobile app” OR “smartphone app” OR “connected health” OR “wearable”). New articles were extracted based on analyzed articles.

### Description of Frameworks

The frameworks were listed in chronological order. Items for each framework were as follows: name of the framework—if none was given, a phrase describing it in the original article was written within quotation marks; publication year; name of the first author, called the *creator* here; country of the creator; organization having funded the creator; health focus or lack of health focus; the target group (ie, population for whom the framework had been initially designed, for example, researchers, health professionals, or software designers); and its design. For the latter, we noted the involvement of the final users (ie, early stage or systematic) and the chain of the development stages (ie, linear, iterative, evolutive, and/or concurrent) [25]. A linear process executes the different stages in a sequential order. An iterative process combines one or more stages before chaining. An evolutive process executes them in a circular pattern in order to obtain, with each revolution, a more mature version of the product. Finally, a parallel process executes one stage or more in a concurrent way. If a framework had evolved through time and had been described by a new publication, the latter was also written in the synthesis table but its creator, as well as the creator’s country and funding organization, remained the original one.

### Categorization of Frameworks and Paradigms

The frameworks were categorized according to the accepted procedure of translational research [26,27]. It may be described as a continuum of steps. It starts with a *prototyping* step, which has a background in engineering, followed by a *mechanisms* step based on a theoretical approach to health behavior change. *Concept* follows, which is a proof-of-concept step regarding health impact based on an exploratory intervention trial. This is followed by a demonstration step, *evidence* of health efficacy and effectiveness, which is based on a confirmatory interventional trial. Finally, a *surveillance* step is implemented for market use based on implementation and dissemination studies.

The frameworks follow three general paradigms: (1) the biomedical paradigm, with its essential phase of clinical research to identify the benefits and risks; (2) the engineering paradigm, with its essential phase to improve the device; and (3) the behavioral paradigm, with its essential phase to evaluate the impact on health behaviors.

## Results

### Overview

The literature review identified 46 frameworks, besides the five-phase drug development model, that met the research criteria (see Table 1).

**Table 1:** Validation and monitoring frameworks of BITs. BIT: behavioral intervention technology.

Frameworks	Authors, year
1. Waterfall model	Royce, 1970 [28]
2. PRECEDE-PROCEED^a^ model	Green, 1974 [29]
3. Prototyping model	Floyd, 1984 [30]
4. Five-phase cancer control model	Greenwald and Cullen, 1985 [31]
5. Flay’s eight-stage health promotion model	Flay, 1986 [32]
6. V life cycle model	Rook, 1986 [33]
7. Spiral life cycle model	Boehm, 1988 [34]
8. Star life cycle model	Harston and Dix, 1989 [35]
9. Rapid application development	Martin, 1991 [36]
10. National Institute on Drug Abuse’s (NIDA) stage model	Onken et al, 1997 [37]
11. Intervention mapping	Bartholomew et al, 1998 [38]
12. Usability engineering life cycle	Mayhew, 1999 [39]
13. Agile software management	Beck et al, 2001 [40]
14. Information technology (IT) implementation framework	Kukafka et al, 2003 [41]
15. Multiphase Optimization STrategy (MOST)	Collins et al, 2005 [42]
16. Framework for evaluating emergent eHealth resources	Pagliari, 2007 [43]
17. Consolidated Standards of Reporting Trials (CONSORT) statement for nonpharmacologic treatments	Boutron et al, 2008 [44] (updated in Boutron et al, 2017 [45])
18. Iterative and incremental model	Cockburn, 2008 [46]
19. Medical Research Council (MRC) complex intervention	Craig et al, 2008 [47]
20. eHealth interventions evaluation process	Catwell and Sheikh, 2009 [48]
21. Center for eHealth Research (CeHRes) roadmap for the development of eHealth technologies	Van Gemert-Pijnen et al, 2011 [49], and Van Velsen et al, 2013 [50]
22. The behavior change wheel	Michie et al, 2011 [51]
23. Consolidated Standards of Reporting Trials of Electronic and Mobile HEalth Applications and onLine TeleHealth (CONSORT-EHEALTH)	Eysenbach et al, 2011 [52], and Eysenbach et al, 2013 [53]
24. mHealth development and evaluation framework	Whittaker et al, 2012 [17]
25. Explore Values, Operationalize and Learn, and eValuate Efficacy (EVOLVE) mixed-methods model	Peterson et al, 2013 [54]
26. Development process of Young and Active	Riiser et al, 2013 [55]
27. It’s LiFe! User-centered design process	Van der Weegen et al, 2013 [56]
28. DoTTI^b^ development framework	Smits et al, 2014 [57]
29. National Institutes of Health (NIH) Stage model	Onken et al, 2014 [58]
30. Behavioral intervention technology model	Mohr et al, 2014 [12]
31. Five-step content validity process	Kassam-Adams et al, 2015 [59]
32. Steps for developing a text-messaging program	Abroms et al, 2015 [60]
33. Person-based approach	Riiser et al, 2013 [55], and Yardley et al, 2015 [61]
34. Obesity-Related Behavioral Intervention Trials (ORBIT) model	Van der Weegen et al, 2013 [56], and Czajkowski et al, 2015 [62]
35. Pragmatic Framework for developing just-in-time adaptive interventions (JITAIs)	Smits et al, 2014 [57], and Nahum-Shani et al, 2015 [63]
36. TElehealth in CHronic disease (TECH) conceptual model	Salisbury et al, 2015 [64]
37. Network for the Improvement of Addiction Treatment (NIATx) model	Gustafson et al, 2016 [65]
38. Integrate, Design, Assess, and Share (IDEAS) framework	Mummah et al, 2016 [66]
39. Chronic disease mHealth app intervention design framework	Wilhide III et al, 2016 [67]
40. Three-phase human-centered design methodology	Harte et al, 2017 [68]
41. DREAM-GLOBAL^c^ framework	Maar et al, 2017 [69]
42. Processes and recommendations for creating mHealth apps for low-income populations	Stephan et al, 2017 [70]
43. Accelerated Creation-To-Sustainment (ACTS) model	Mohr et al, 2017 [71]
44. User-centered design process	Vilardaga et al, 2018 [72]
45. Eight-step scoping framework	Davidson et al, 2019 [73]
46. Targeting, Understanding, Designing, Evaluating, and Refining (TUDER) framework	Wang et al, 2019 [74]

^a^PRECEDE-PROCEED: Predisposing, Reinforcing, and Enabling Constructs in Educational Diagnosis and Evaluation—Policy, Regulatory, and Organizational Constructs in Educational and Environmental Development.

^b^DoTTI: Design and develOpment, Testing early iterations, Testing for effectiveness, Integration and implementation.

^c^DREAM-GLOBAL: Diagnosing hypeRtension—Engaging Action and Management in Getting LOwer Bp in Aboriginal and LMIC (lower- and middle-income countries).

### Description of Frameworks

#### Frameworks

The results showed that, out of 46 frameworks, 2 (4%) were designed in the 1970s, 6 (13%) in the 1980s, 4 (9%) in the 1990s, 8 (17%) in the 2000s, and 26 (57%) in the 2010s (see Multimedia Appendix 1). Their creators were mainly from the United States (24/46, 52%) and the United Kingdom (10/46, 21%). The Netherlands, Germany, Canada, Australia, Brazil, France, Ireland, Norway, and New Zealand were the remaining creators’ countries. A total of 8 frameworks out of 46 (17%) were created by private software company workers. A total of 4 frameworks out of 46 (9%) were designed by a national or a supranational public organization: the National Institutes of Health (NIH), the Medical Research Council (MRC), the National Health Service (NHS), and the Consolidated Standards of Reporting Trials (CONSORT). Universities were the main institutions (34/46, 74%), with 16 out of 46 (35%) from the United States. A total of 29 out of 46 frameworks (63%) were supported by a research grant: out of 46 frameworks, 29 (63%) were funded by a public grant, 12 (26%) were funded by the NIH, and 6 (13%) were funded by the English public health system. Out of 46 frameworks, 5 (11%) were funded by universities, 3 (7%) were funded by the private sector, and 3 (7%) were funded by a public-private partnership.

#### Purpose and Target Population

A total of 36 frameworks out of 46 (78%) were created with an individual health focus. Among them, the NIH Stage model was created to be relevant for clinical sciences [58] and the CONSORT statement was created for nonpharmacologic treatments for all nonpharmacological interventions [44]. Out of 46 frameworks, 3 (7%) aimed to validate health promotion programs [29,32,38]. A total of 23 of 46 (50%) were targeted to validate health behavior change interventions: of these 23, 15 (65%) were aimed at eHealth interventions in general [12,41,43,48,49,52,59,61,64-66,71-74], 2 (9%) at Internet interventions [55,57], and 6 (26%) at mobile interventions [17,56,60,67,69,70]. Among the 8 frameworks out of 23 (35%) aimed at health behavior change that were not focused on eHealth, 5 (63%) were created for behavior change in general [42,47,51,54,63] and 3 (38%) were dedicated to diseases such as cancer [31], addiction [37], and all chronic disease [62]. A total of 10 out of 46 frameworks (22%) were created with an original purpose that was not associated with health, but was associated with software development [28,33,34,36,40,46], human-computer interfaces [35,39], or engineering in general [30,68].

Out of 46 frameworks, 17 (37%) were designed explicitly for intervention designers: 11 (24%) for software designers [17,30,33,35,36,39,43,46,48,49,64], 1 of which was for software evaluators [66], and 6 (13%) for designers of health behavior change interventions [12,41,51,61-63], 2 of which were based on technologies to change health behaviors [12,41]. A total of 9 out of 46 frameworks (20%) were aimed at researchers; these included researchers in general [54,58], scientific paper authors or scientific journal editors [44,51], researchers and stakeholders [32,71], researchers and industry professionals [66], and health researchers and software designers [43,60]. Finally, 13 frameworks out of 46 (28%) were intended for all stakeholders: professionals, researchers, users, clinicians, and other health helpers [42,46,55-57,59,65,67,68-70,72,73]. In 6 out of 46 cases (13%), the target of the framework was not mentioned; however, the reading of these articles was directed toward software designers [28,34,40], researchers, stakeholders, and health program planners [29,31,37]. Out of 46, 25 frameworks (54%) were created for software designers and 24 (52%) were created for researchers.

#### Organization

In 28 out of 46 frameworks (61%), the final user was involved early and systematically (ie, at each step). In 27 of them (59%), he or she played an active role in the BIT’s specification and assessment [17,35,36,39,40,43,48,49,54-57,59-62,64-74]. In the V life cycle model, the role was only as an evaluator [33].

A total of 2 frameworks out of 46 (4%) had a linear sequence in their phases. The Waterfall model adopts a seven-phase structure: identification of the system specifications, identification of the software specifications, analysis, program design, coding, tests, and operations [28]. Inspired by the latter, the V life cycle model has a V-shaped structure, which matches development and testing phases and involves clients, followed by final users, in the development [33]. In addition to the linear sequence, 17 out of 46 frameworks (37%) had an iterative structure [12,17,31,32,42,44,52,54-56,61,62,66,69,70,72,73], allowing a refining step at each phase in the case of suboptimal results. The iterative structure is one of the assets used by the Multiphase Optimization STrategy (MOST) and the Obesity-Related Behavioral Intervention Trials (ORBIT) model frameworks to optimize interventions [42,62]. A total of 15 out of 46 frameworks (33%) were also associated with an evolutive process, creating a cyclic organization to improve the product progressively [30,34,35,37-39,43,47-49,51,58,60,67,68]. Finally, 11 out of 46 frameworks (24%) also integrated a parallel process [29,36,40,41,46,57,59,64,65,71,74]; 1 of those was the Agile software management framework, which aims for a high degree of adaptability to satisfy the clients [40].

### Framework Categorization

**Figure 1:**
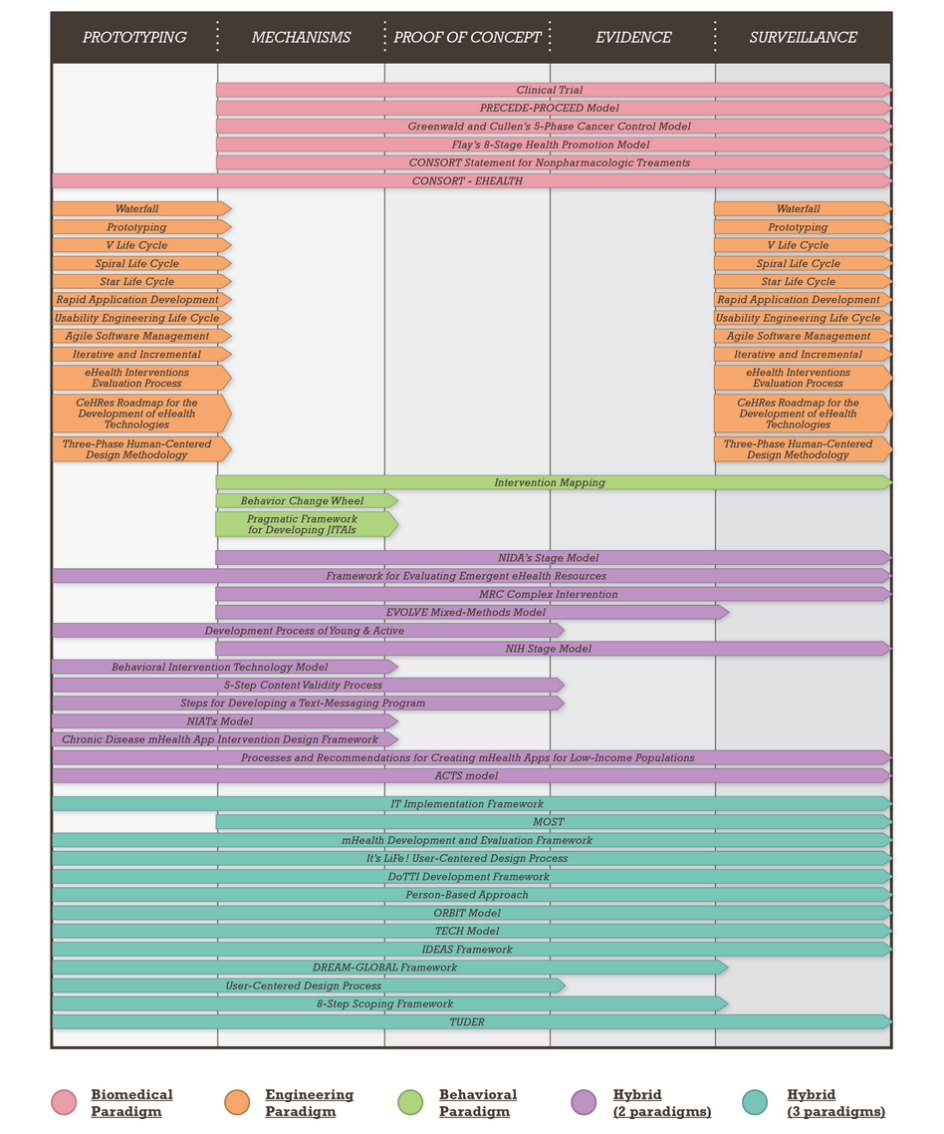
Proposed frameworks to validate and monitor behavioral intervention technologies (BITs). ACTS: Accelerated Creation-To-Sustainment; CeHRes: Center for eHealth Research; DoTTI: Design and develOpment, Testing early iterations, Testing for effectiveness, Integration and implementation; DREAM-GLOBAL: Diagnosing hypeRtension—Engaging Action and Management in Getting LOwer Bp in Aboriginal and LMIC (lower- and middle-income countries); EVOLVE: Explore Values, Operationalize and Learn, and eValuate Efficacy; IDEAS: Integrate, Design, Assess, and Share; IT: information technology; JITAI: just-in-time adaptive intervention; MOST: Multiphase Optimization STrategy; MRC: Medical Research Council; NIATx: Network for the Improvement of Addiction Treatment; NIDA: National Institute on Drug Abuse; NIH: National Institutes of Health; ORBIT: Obesity-Related Behavioral Intervention Trials; PRECEDE-PROCEED: Predisposing, Reinforcing, and Enabling Constructs in Educational Diagnosis and Evaluation—Policy, Regulatory, and Organizational Constructs in Educational and Environmental Development; TECH: TElehealth in CHronic disease; TUDER: Targeting, Understanding, Designing, Evaluating, and Refining.

The categorization of frameworks, according to their coverage of the continuum of steps, is shown in Figure 1.

The Waterfall model is the only one that covers only the prototyping step [28]. Other frameworks cover the prototyping and surveillance steps by integrating the final user [30,33-36,39,40,46,48,49,68].

The behavior change wheel and the pragmatic framework for developing just-in-time adaptive interventions (JITAIs) are focused on the mechanisms step [51,63]. The BIT model, the chronic disease mHealth app intervention design framework, and the Network for the Improvement of Addiction Treatment (NIATx) model cover the prototyping and mechanisms steps by associating the conception of both numeric and behavioral interventions [12,17,65].

The Explore Values, Operationalize and Learn, and eValuate Efficacy (EVOLVE) and MOST frameworks cover the mechanisms, concept, and evidence steps. A total of 9 frameworks out of 46 (20%) are extended over the mechanisms, concept, evidence, and surveillance steps [29,31,32,37,44,47,49,58,62]. Finally, the whole continuum of steps is covered by 12 out of 46 (26%) frameworks [17,41,43,52,56,57,61,64,66,70,71,74].

### Paradigm Categorization

Figure 1 illustrates the categorization of frameworks based on their paradigms. Most belonged to one paradigm (ie, biomedical, engineering, or behavioral), while few referred to two or three paradigms.

Out of 46 frameworks, 12 (26%) fell within the paradigm of engineering [28,30,33-36,39,40,46,48,49,68] and 3 (7%) were based on the behavioral paradigm [38,51,63]. A total of 9 (20%) frameworks were based on these two latter paradigms [12,55,59,60,65,67,70-72]. A total of 5 (11%) frameworks were based on the biomedical paradigm, which draws on the clinical trial, specifically the randomized controlled trial [28,31,32,44,52]. A total of 4 others (9%) also incorporated a theoretical approach of behavior change [37,47,54,58]. The framework for evaluating emergent eHealth resources was based on the biomedical and engineering paradigms [43]. A total of 12 (26%) frameworks mixed three paradigms [17,41,42,56,57,61-63,67,69,73,74].

## Discussion

### Principal Findings

Our narrative review of the literature showed the absence of a unique and consensual validation and surveillance framework for health behavior intervention technology. This conclusion is in keeping with the statement of Bradway et al about mHealth assessment: “too many initiatives, too few answers” [75]. To date, 46 frameworks coexist, besides the five-phase drug development model, without a predominance of, or a convergence toward, one of them. Their number has increased exponentially over time since the 1970s. The United States and the United Kingdom are the main countries that have proposed models, probably due to their university research productivity and new-technology industry leadership [76]. They are motivated, on the one hand, by the plethoric and easily available offer of digital tools and networks and, on the other hand, by the failure to prevent unhealthy diet, addiction, and physical inactivity [2].

Beyond the quantitative increase of these frameworks, this review underlines an increased heterogeneity. One surprising finding is that the benefit-to-risk ratio is not a main goal. Contrary to drug claims regarding societal recognition and reimbursement for their impact on health outcomes [77], showing evidence of safety and efficacy is paradoxically not a priority for BITs. This fact can explain why researchers have noted that medical mobile phone apps are lightly used by patients in the medium term, have little involvement from health professionals, lack characterization of their content, and have a low integration of behavior change theories [78]. The same problem can be found for connected health devices. The lack of evidence of their efficacy leads to their nonprescription by general practitioners, who picture them mainly as a supplementary organizational constraint [79]. Most frameworks are inspired by the paradigms of engineering and behavioral sciences. Engineering is focused on technicity (ie, release criteria, ergonomics, user experience, and data security), which leads to uncertainties about these technologies’ health contributions and turns them into entertainment products or gadgets [80]. The behavioral sciences bring an understanding of behavior change mechanisms and skills techniques without determining and comparing their health impacts [81,82]. This explains how translational research is unusual in this area compared to that for drugs [83]. To date, for instance, there is no validation model for psychotherapies [84].

Beyond the quantitative increase and diversification of frameworks, we have noted the emergence of common principles. The first one is an assumed superiority with respect to health and health behavior change of some BITs compared to others. This hypothesis aims to address the plethoric and exponential offer of digital solutions sustained by marketing. This growing need to know the efficacy of each BIT and to compare them to other solutions to change health behaviors comes from both health professionals, who seek to recommend or even prescribe evidence-based solutions, and health users, who seek solutions beyond marketing and/or users’ experiences from social networks.

The second is the requirement of user involvement during the beginning of BIT development and after market access. This principle exists in every recent framework based on the behavioral science, engineering, or biomedical paradigms. It requires a coconstruction with the user and considers his or her experience. It involves the end user through a user-centered design from engineering [85,86]. It requires a patient-centered approach to the provision of personalized care in the biomedical area [87]. The patient’s preferences are considered and may allow improved chances of stable and efficient use.

The third is the ambition to evaluate BITs beyond simple satisfaction. This is more or less oriented by manufacturers to aim at effectiveness in medicine becoming more and more predictive, preventive, personalized, and participative; this is called 4P medicine [88,89].

The fourth is the shortening of the delay for upstream validation, until its complete suppression in some engineering frameworks, and a multiplication of the downstream surveillance methods, such as implementation studies, experience feedback (eg, to a learned society or health authority), and big data analyses combining multimodal data. The shortening of the upstream period is linked, on the one hand, to a supposedly low dangerousness of these health solutions and, on the other hand, to the short life cycle of the technologies involved [90]. The development time of a drug, from the lab bench to its marketing authorization, is between 17 and 24 years [91-93]. It is incompatible with digital innovation. Digital industrialists assume that hundreds of millions of users will allow for the evaluation of BITs’ effectiveness on a large scale thanks to big data analyses [94,95].

The fifth is the introduction of hybrid frameworks integrating the development and updating processes from engineering, behavior change theories, techniques from behavioral sciences, and the rigorous approach of validation from the biomedical area (eg, the FDA and the Environmental Management Association). This hybridization contributes to the creation of interventions standing between medical devices and products in the category of *general commodities*. The scientific process to develop and validate them, the rules for marketing authorization, and surveillance have not yet been defined for a country or a continent and are still under construction. Many digital industrialists wish to avoid the validation process for medical devices in view of its constraints and costs; at the same time, they aim to indicate to health professionals and users the health impact of their solutions. They are encouraged to address this by mutual insurances, other insurances, and banks.

We stress two extreme positions for the studied frameworks that may become problematic for the development of BITs in the health sector. The first is that of permanent technological innovation. It is embodied in the Agile software management framework. Flexibility, based on user demand, dictates each evolution. Health is a market like any other. If this approach was to win in this area, for example, to make substantial savings in clinical research, no counterpart could be asked for, concerning health benefits. Big data analyses will not guarantee a relevant comparability between BITs [96]. The second problem at the opposite extreme is that marketing authorization of the BIT must be conditional on the completion of a well-designed randomized controlled trial. This process has been a part of the success of drugs in the last century. These trials are long, costly, and debatable in terms of methodology for BITs, if only for the choice of control group [97].

### Limitations and Strengths

To identify every validation and surveillance framework of BITs is challenging. This area is new, it is based on a number of different paradigms, and develops in an uncontrolled way from one country to another and from one industry to another. One of the identified difficulties within these publications is the lack of common terminology [98,99]. The originality of our review is the spanning of the three approaches (ie, engineering, behavioral sciences, biomedical sciences). Although they are often opposed, the concern here was to note the borrowings and contributions that they could mutually bring through transversal thinking.

### Conclusions

Validating a BIT for health has the following specific challenges: achieve an efficient and quick development; understand and promote long-term adherence; be supported by behavior change theories and techniques; evaluate effectiveness and cost-effectiveness; and ensure rigorous management in terms of regulation, ethics, and information [93]. Patient expectations are relayed through the media and through the arrival on the health market of digital players proposing short life-cycle products due to rapid obsolescence of technology; this justifies the need for a consensual validation and surveillance framework for nonpharmacological interventions. This framework will necessarily differ from that used for drugs. Our review has identified 46 frameworks, none of which dominates for BITs. Three paths are opened: (1) the anarchic continuous development of new competing frameworks that prevents any convergence in a standardized validation and surveillance framework, with its consequential recognition by health authorities; (2) the movement toward the type of framework for drug evaluation centered on establishing its effectiveness before marketing authorization to guarantee its safety for users, which is heavy and costly; and (3) the implementation of a framework reliant on big data analysis, based on a posteriori research and an autoregulation of a market; however, that does not address the safety risk for the health user, as the market will not regulate safety or efficacy issues. This article calls for a minimal upstream clinical phase and an increased surveillance of BITs to address the risk of semantic amalgams, inappropriate prescriptions, and induced misuses [16,100]. A BIT cannot be a simple tool but must be a complex strategy integrated in a given environment [101]. A BIT must make sense by virtue of its interaction with the context, building a system, and must be evaluated as such [100].

## Appendix

Multimedia Appendix 1 Table of frameworks.

## Abbreviations

ACTS: Accelerated Creation-To-Sustainment
BIT: behavioral intervention technology
CeHRes: Center for eHealth Research
CEPS: Plateforme universitaire Collaborative d’Evaluation des programmes de Prévention et de Soins de support
CONSORT: Consolidated Standards of Reporting Trials
CONSORT-EHEALTH: Consolidated Standards of Reporting Trials of Electronic and Mobile HEalth Applications and onLine TeleHealth
DoTTI: Design and develOpment, Testing early iterations, Testing for effectiveness, Integration and implementation
DREAM-GLOBAL: Diagnosing hypeRtension—Engaging Action and Management in Getting LOwer Bp in Aboriginal and LMIC (lower- and middle-income countries)
EVOLVE: Explore Values, Operationalize and Learn, and eValuate Efficacy
FDA: US Food and Drug Administration
IDEAS: Integrate, Design, Assess, and Share
IT: information technology
JITAI: just-in-time adaptive intervention
JMIR: Journal of Medical Internet Research
MOST: Multiphase Optimization STrategy
MRC: Medical Research Council
NHS: National Health Service
NIATx: Network for the Improvement of Addiction Treatment
NIDA: National Institute on Drug Abuse
NIH: National Institutes of Health
ORBIT: Obesity-Related Behavioral Intervention Trials
PRECEDE-PROCEED: Predisposing, Reinforcing, and Enabling Constructs in Educational Diagnosis and Evaluation—Policy, Regulatory, and Organizational Constructs in Educational and Environmental Development
SMS: short message service
TECH: TElehealth in CHronic disease
TUDER: Targeting, Understanding, Designing, Evaluating, and Refining
WHO: World Health Organization
