# Supplementary material for: Identifying Frameworks for Validation and Monitoring of Consensual Behavioral Intervention Technologies: Narrative Review
Source: J Med Internet Res. 2019 Oct 16;21(10):e13606. doi: 10.2196/13606 (PMC6822061; doi:10.2196/13606)
Supplement: Multimedia Appendix 1 [file jmir_v21i10e13606_app1.pdf]

Multimedia Appendix 1: Table of frameworks.

Framework	Authors	Publication year	Biblio	Creator's Country	Organization having funded the creator	Organisation having funded the creation of the framework itself	Focus	Target group	Design process (Linear, Iterative, Evolutive, Parallel, Incremental	Early Implication of end users in the process?	Systematic Implication of end users in the process?	Categorization of the frameworks: P-MCES	Categorization of the paradigms
Waterfall model	Royce	1970	Royce WW. Managing the development of large software systems. In: proceedings of IEEE WESCON. Los Angeles; 1970. p. 328–338	USA	Lockheed Software Technology Center	None	Non-Health (software development)	Not specified (software designers / development managers)	Linear	No	No	P	Engineering paradigm
PRECEDE-PROCEED model	Green	1974	Green LW. Toward cost-benefit evaluations of health education: some concepts, methods and examples. Health Education Monograph 1974;2(Suppl 2):34-64.	USA	School of Hygiene and Public Health, John Hopkins University	None	Health (Health promotion programs)	Not specified (searchers, policy makers, health programs planners)	Iterative, Evolutive, Parallel	No	No	MCES	Biomedical model
Prototyping model	Floyd	1984	Floyd C. A systematic look at prototyping, in: Budde, R., Kuhlenkamp, K., Mathiassen, L. and Zullighoven, H. Approaches to Prototyping, Springer-Verlag: Heidelberg, 1-17.1984.	Germany	Institut d'informatique appliquée	None	Non-Health (engineering)	Software designers / development managers	Iterative, Evolutive	No	No	P-S	Engineering paradigm
Greenwald and Cullen's	Greenwald &	1985	Greenwald P, Cullen JW. The new	USA	Division of Cancer	None	Health (Behavior	Not specified (searchers, health	Iterative	No	No	MCES	Biomedical model

5-phase cancer control model	Cullen		emphasis in cancer control. Journal of the National Cancer Institute. 1985;74(3):543–551. 1985.		Prevention and Control, Cancer Institute, NIH		change, cancer)	policy makers)					
Flay's 8-stage health promotion model	Flay	1986	Flay BR. Efficacy and effectiveness trials (and other phases of research) in the development of health promotion programs. Prev Med. ;15(5):451 74. 1986.	USA	Health Bahavior Research Institute, University of Southern California	NIH	Health (Health promotion programs)	Searchers, health policy makers	Iterative	No	No	MCES	Biomedical model
V life cycle model	Rook	1986	Rook P. Controlling software projects. Software Engineering Journal - Controlling software projects, vo.1, no.1, pp.7-16, 1986.	UK	GEC Software	None	Non-Health (software development)	Software designers / development managers	Linear	Y (evaluation)	Y (evaluation only)	P-S	Engineering paradigm
Spiral life cycle model	Boehm	1988	Boehm BW. A spiral model of software development and enhancement. Computer. mai 1988;21(5):61-72	USA	TRW Defense Systems Group	None	Non-Health (software development)	Not specified (software designers / development managers)	Iterative, Evolutive	No	No	P-S	Engineering paradigm
Star life cycle model	Harston & Dix	1989	Harston HR, Hix D. `Towards empirically derived methodologies and tools for HCI development. *International Journal of Man-Machine Studies*, 1989;31:477-494.	USA	Department of Cumputer Science, Virginia Polytechnic Institute and State University	National Science Foundation, IBM Federal Systems Division, the Software Productivity Consortium and the Virginia Center for Innovative Technology	Non-Health (human-computer interface)	Software designers / development managers	Iterative, Evolutive	Y (analysis and evaluation)	Y (analysis and evaluation	P-S	Engineering paradigm
Rapid application development	Martin	1991	Martin J. Rapid Application Development. Macmillan. 1991	UK	IBM	None	Non-Health (software development)	Designers, software development managers	Iterative, Evolutive , Parallel	Y (analysis and evaluatio	Y (analysis and evaluatio	P-S	Engineering

			(LIVRE)							n)	n)		
NIDA's stage model	Onken *et al.*	1997	Onken LS, Blaine JD, Battjes RJ. Behavioral therapy research: A conceptualization of a process. In:Henggeler SW, Santos AB,editors. Innovative approaches for difficult-to-treat populations. Washington,D.C.: American Psychiatric Press, Inc. p.477-85. 1997.	USA	National Institute on Drug Abuse (NIH)	NIH	Health (Behavior change,drug abuse)	Not specified (researchers, health policy makers)	Iterative, Evolutive	No	No	MCES	Biomedical model, Psychological paradigm
Intervention mapping	Bartholomew *et al.*	1998	Bartholomew LK, Parcel GS, Kok G. Intervention mapping: a process for developing theory- and evidence-based health education programs. Health Educ Behav. oct 1998;25(5):545-63.	USA	Center for Health Promotion Research and Development, University of Tewas Health Science Center	None	Health (health promotion programs)	Health education program planners	Iterative, Evolutive	No	No	MCES	Behavioral theory
Usability Engineering Life Cycle	Mayhew	1999	Mayhew D. The usability engineering lifecycle: a practitioner's handbook for user interface design (LIVRE)	USA	Northeastern university	None	Non-Health (human-computer interface)	Designers, software development managers	Iterative, Evolutive	Y (analysis and evaluation)	Y (analysis and evaluation)	P-S	Engineering paradigm
Agile software management	Beck *et al.*	2001	Beck K, Beedle M, Bennekum A, Cockburn A, Cunningham W, Fowler M, Grenning J, Highsmith J, Hunt A, Jeffries R, Kern J, Marick B, Martin R, Mellor S, Schwaber K, Sutherland J, Thomas D.	USA	Chrysler/Facebook	None	Non-Health (software development)	Not specified (software designers / development managers)	Iterative, Evolutive , Parallel	Y (analysis and evaluation)	Y (analysis and evaluation)	P-S	Engineering paradigm

			Manifesto for Agile software development. www.agilemanifesto.org. 2001										
IT implementation framework	Kukafka *et al.*	2003	Kukafka R, Johnson SB, Linfante A, Allegrante JP. Grounding a new information technology implementation framework in behavioral science: a systematic analysis of the literature on IT use. J Biomed Inform. 2003;36(3):218-27.	USA	Department of sociomedical sciences, Joseph Mailman School of Public Heamth, Columbia University	None	Health (Behavior change, ehealth)	Behavior change intervention designers using information technologies	Iterative, Evolutive , Parallel	No	No	P-MCES	Biomedical model, Psychological paradigm, Engineering paradigm
Multiphase Optimization STrategy (MOST)	Collins et *al.*	2005	Collins LM, Murphy SA, Nair VN, Strecher VJ. A strategy for optimizing and evaluating behavioral interventions. Annals of Behavioral Medicine. 2005;30(1):65–73.	UK	Methodology Center, Department of Human Development and Family Studies, Pennsylvania State University	NIH, National Science Foundation	Health (Behavior change)	Intervention researchers, intervention targets, health care providers and other stakeholders	Iterative	No	No	MCE	Biomedical model, Psychological paradigm, Engineering paradigm
"Framework for evaluating emergent eHealth ressources"	Pagliari	2007	Pagliari C. Design and evaluation in eHealth: challenges and implications for an interdisciplinary field. J Med Internet Res. 2007;9(2):e15.	UK	EHealth Interdisciplinary Research Rroup, University of Edinburgh	None	Health (Behavior change, eHealth)	Software developers and health services researchers	Iterative, Evolutive	Y (analysis and evaluation)	Y (analysis and evaluation)	P-MCES	Biomedical model, Engineering paradigm
CONSORT Statement for Nonpharmacologic Treaments	Boutron *et al.*	2008	Boutron I, Moher D, Altman DG, Schulz KF, Ravaud P. Methods and processes of the CONSORT Group: example of an extension for trials assessing nonpharmacologic	France	Department of Biostatistical Epidemiology and Clinical Research, INSERM U738, Université Paris 7 Denis Diderot/Bichat	Département de la Recherche Clinique et du Développement, Assistance Publique des Hôpitaux de	Health (Nonpharmacologic treatments)	Researchers (Journal authors), journal editors	Iterative	No	No	MCES	Biomedical model

			treatments. Annals of Internal Medicine. 2008;148(4):W–60.		-Claude Bernard Hospital	Paris; Department of Biostatistical Epidemiology and Clinical Research, INSERM U738, Université Paris 7 Denis Diderot/Bichat-Claude Bernard Hospital							
Iterative and incremental model	Cockburn	2008	Cockburn A. Using both incremental and iterative developement STSC Cross Talk, 21(5),27-30. 2008	USA	Companies (IBM)	None	Non-Health (Software development)	Development teams, development managers	Iterative, Evolutive , Parallel	No	No	P-S	Engineering paradigm
MRC Complex intervention	Craig *et al.*	2008	Craig, P., Dieppe, P., Macintyre, S., Michie, S., Nazareth, I., Petticrew, M. Developing and evaluating complex interventions: the new Medical Research Council guidance. British Medical Journal. 2008;337, a1655	UK	Medical Research Council Population Health Sciences Research Network	Medical Research Council Health Services and Public Health Research Board	Health (complex interventions)	Researchers, research funders, policy makers, practitioners and other commissioners and users of evaluation	Iterative, Evolutive	No	No	MCES	Biomedical model, Psychological paradigm
"EHealth interventions evaluation process"	Catwell & Sheikh	2009	Catwell L, Sheikh A. Evaluating eHealth interventions: the need for continuous systemic evaluation. PLoS Med. 2009;6(8):e1000126	UK	Center for Population Health Sciences, University of Edinburgh	NHS Connecting for Health Evaluation Programme	Health (Behavior change, ehealth)	Information Communication Technology developers	Iterative, Evolutive	Y (analysis and evaluation)	Y (analysis and evaluation)	P-S	Engineering paradigm
Center for eHealth Research (CeHRes)	Van Gemert-Pijnen et *al.*	2011	van Gemert-Pijnen JE, Nijland N, van Limburg M, Ossebaard HC,	Netherlands	Department of Psychology, Health and Technology/C	None	Health (Behavior change, eHealth)	ehealth developers	Iterative, Evolutive	Y (analysis and evaluatio	Y (analysis and evaluatio	P-S	Engineering paradigm

roadmap for the developement of eHealth technologies			Kelders SM, Eysenbach G, et al. A Holistic Framework to Improve the Uptake and Impact of eHealth Technologies. Journal of Medical Internet Research. 13 déc 2011;13(4):e111.		enter for eHealth Research and Disease Management, Faculty of Behavioural Sciences, University of Twente					n)	n)		
The Behavior Change Wheel	Michie et *al.*	2011	Michie S, van Stralen MM, West R. The behaviour change wheel: a new method for characterising and designing behaviour change interventions. Implement Sci. 2011;6:42.	UK	Research Department of Clinical, Educational, and Health Psychology, University College London.	Cancer Research UK	Health (Behavior change)	Behavior change intervention designers	Iterative, Evolutive	No	No	M	Psychological paradigm
CONSORT ehealth	Eysenbach *et al.*	2011	Eysenbach G, Group C-E. CONSORT-EHEALTH: improving and standardizing evaluation reports of Web-based and mobile health interventions. J Med Internet Res. 2011;13(4):e126. Eysenbach G. CONSORT-EHEALTH: implementation of a checklist for authors and editors to improve reporting of web-based and mobile randomized controlled trials. Stud Health Technol Inform. 2013;192:657-61.	Canada	University Health Network, Center for Global eHEalth Innovation & Techna Institute, Toronto/ Institute for Health Policy, Management, and Evaluation, University of Toronto/ JMIR Publications	None	Health (Behavior change, ehealth)	Researchers (Journal authors), journal editors	Linear, Iterative	No	No	P-MCES	Biomedical model

mHealth Development and Evaluation Framework	Whittaker *et al.*	2012	Whittaker R, Merry S, Dorey E, Maddison R. A Development and evaluation process for mHealth interventions: Examples from New Zealand. J Health Com 2012;17:11-21.	New Zealand	Clinical Trials Research Unit, University of Auckland	the Health Research Council of New Zealand, Oakley Mental Health Foundation, University of Auckland, Digital Strategy Community Partnership Fund, Vodafone New Zealand Ltd, Auckland UniServices Ltd, National Heart Foundation, Cancer Society of New Zealand, Alcatel.	Health (Behavior change,mHealth)	Not specified (mHealth intervention developers and/or evaluators)	Iterative	Y (analysis and evaluation)	Y (analysis and evaluation)	P-MCES	Biomedical model,Psychological paradigm, Engineering paradigm
Explore Values, Operationalize and Learn, and eValuate Efficacy (EVOLVE) mixed-methods model	Peterson *et al.*	2013	Peterson JC, Czajkowski S, Charlson ME, Link AR, Wells MT, Isen AM, et al. Translating basic behavioral and social science research to clinical application: The EVOLVE mixed methods approach. Journal of Consulting and Clinical Psychology. 2013;81(2):217-30.	USA	Division of Clinical Epidemiology and Evaluative Sciences Research, Department of Medicine, and Center for Integrative Medicine, Weill Cornell Medical College	NIH	Health (Behavior change)	Researchers	Iterative	Y (analysis and evaluation)	Y (analysis and evaluation)	MCE	Biomedical model, Psychological paradigm
Development process of	Riiser *et al.*	2013	Riiser K, Løndal K, Ommundsen Y,	Norway	Oslo and Akershus	Norwegian ExtraFounda	Health (internet	Researchers, designers,	Iterative	Y (analysis	Y (analysis	P-MC	Psychological paradigm,

Young & Active			Sundar T, Helseth S. Development and Usability Testing of an Internet Intervention to Increase Physical Activity in Overweight Adolescents. JMIR Res Protoc 2013;2(1):e7		University College of Applied Sciences, Oslo	tion for Health and Rehabilitation	intervention, increase physical activity, overweight adolescents)	developers, and representatives from the target group		and evaluation)	and evaluation)		Engineering paradigm
It’s LiFe! User-centered design process	Van der Weegen *et al.*	2013	Van der Weegen S, Verwey R, Spreeuwenberg M, Tange H, van der Weijden T, de Witte L. The development of a mobile monitoring and feedback tool to stimulate physical activity of people with a chronic disease in primary care: a user-centered design. JMIR Mhealth Uhealth. 2013;1(2):e8.	Netherlands	CAPHRI School for Public Health and Primary Care, Departement of Health Services Research, Maastrich University	Netherlands Organization for Health Research and Development	Health (Behavior change, chonic disease, ehealth)	Researchers	Iterative	Y (analysis and evaluation)	Y (analysis and evaluation)	P-MCES	Biomedical model, Psychological paradigm, Engineering paradigm
DoTTI Development Framework	Smits *et al.*	2014	Smits R, Bryant J, Sanson-Fisher R, Tzelepis F, Henskens F, Paul C, et al. Tailored and integrated Web-based tools for improving psychosocial outcomes of cancer patients: the DoTTI development framework. J Med Internet Res. 2014;16(3):e76.	Australia	Priority Research Centre for Health Behaviour, University of Newcastle & Hunter Medical Research Institute, Callaghan	New South Wales Cancer Institute Translational Health Services, Cancer Council NSW, Australian Research Council	Health (Behavior change, ehealth, cancer)	Stakeholders (including researchers, clinicians, consumers, and programmers)	Iterative, Evolutive , Parallel	Y (analysis and evaluation)	Y (analysis and evaluation)	P-MCES	Biomedical model, Psychological paradigm, Engineering paradigm
NIH Stage model	Onken *et al.*	2014	Onken LS, Carroll KM, Shoham V, Cuthbert BN, Riddle M. Reenvisioning Clinical Science:	USA	National Institute on Drug Abuse (NIH)	NIH	Health (Clinical science)	Researchers and students to learn how to conduct research in every domain of clinical	Iterative, Evolutive	No	No	MCES	Biomedical model, Psychological paradigm

			Unifying the Discipline to Improve the Public Health. Clin Psychol Sci. 2014;2(1):22-34.					science					
Behavioral Intervention Technology Model	Mohr *et al.*	2014	Mohr DC, Schueller SM, Montague E, Burns MN, Rashidi P. The Behavioral Intervention Technology Model: An Integrated Conceptual and Technological Framework for eHealth and mHealth Interventions. Journal of Medical Internet Research. 5 juin 2014;16(6):e146.	USA	Center for Behavioral Intervention Technologies, Department of Preventive Medicine, Northwestern University	NIH	Health (Behavior change, ehealth)	Designers, software development managers	Iterative	No	No	P-M	Psychological paradigm, Engineering paradigm
5-Step Content Validity Process	Kassam-Adams *et al.*	2015	Kassam-Adams N, Marsac ML, Kohser KL, Kenardy JA, March S, Winston FK. A New Method for Assessing Content Validity in Model-Based Creation and Iteration of eHealth Interventions. J Med Internet Res 2015;17(4):e95	USA	University of Pennsylvania, Philadelphia	Eunice Kennedy Schriver National Institute of Child Health and Human Development	Health (eHealth)	Researchers, Developers, users	Iterative, Evolutive , Parallel	Y (analysis and evaluation)	Y (analysis and evaluation)	P-MC	Psychological paradigm, Engineering paradigm
Steps for developing a text messaging program	Abroms *et al.*	2015	Abroms LC, Whittaker R, Free C, Mendel Van Alstyne J, Schindler-Ruwisch JM. Developing and Pretesting a Text Messaging Program for Health Behavior Change: Recommended Steps. JMIR	USA	The Milken Institute School of Public Health, The George Washington University, Washington, DC; National Institute for Health Innovation,	None	Health (Behavior change, text-messaging program)	Designers, Researchers	Interative, Evolutive	Y (analysis and evaluation)	Y (analysis and evaluation)	P-MC	Psychological paradigm, Engineering paradigm

			Mhealth Uhealth. 2015;3(4):e107.		The University of Auckland; Clinical Trials Research Unit, London School of Hygiene and Tropical Medicine, London								
Person-based approach	Yardley *et al.*	2015	Yardley L, Morrison L, Bradbury K, Muller I. The Person-Based Approach to Intervention Development: Application to Digital Health-Related Behavior Change Interventions. Journal of Medical Internet Research. 30 janv 2015 ;17(1) :e30.	UK	Department of Psychology, Faculty of Social and Human Sciences, University of Southampton	Engineering and Physical Sciences Research Council	Health (Behavior change, ehealth)	Behavior change intervention designers	Iterative	Y (analysis and evaluation)	Y (analysis and evaluation)	P-MCES	Biomedical model, Psychological paradigm, Engineering paradigm
ORBIT Model	Czajkowski *et al.*	2015	Czajkowski, S.M., Powell, L.H., Adler, N., Naar-King, S., Reynolds, K.D., Hunter, C.M., Laraia, B., Olster, D.H., Perna, F.M., Peterson, J.C., Epel, E., Boyington, J.E., Charlson, M.E. (2015). From Ideas to Efficacy: The ORBIT model for developing behavioral treatments for chronic diseases. Health Psychology, 10, 971-982.	USA	National Heart, Lung, and Blood Institute,NIH	NIH	Health (Behavior change, chronic diseases)	Behavior change intervention designers	Iterative	Y (analysis and evaluation)	Y (analysis and evaluation)	MCES	Biomedical model, Psychological paradigm, Engineering paradigm
Pragmatic Framework for	Nahum-Shani et *al.*	2015	Nahum-Shani I, Hekler EB, Spruijt-Metz D. Building	USA	Institute for Social Research,	NIH	Health (Behavior change, JITAI)	Behavior change intervention designers (JITAI)	Iterative, Evolutive	No	No	M	Psychological paradigm

Developing JITAIs			health behavior models to guide the development of just-in-time adaptive interventions: A pragmatic framework. Health Psychology. 2015 ;34(Suppl) :1209-19.		University of Michigan								
Telehealth in Chronic disease (TECH) conceptual model	Salisbury et *al.*	2015	Salisbury C, Thomas C, O'Cathain A, Rogers A, Pope C, Yardley L, et al. Telehealth in Chronic disease: mixed-methods study to develop the TECH conceptual model for intervention design and evaluation. BMJ open. 2015;5(2):e006448.	UK	University of Bristol, Centre for Academic Primary Care, School of Social and Community Medicine	National Institute for Health Research (NIHR)	Health (Behavior change, ehealth)	Designers and evaluators of telehealth programmes	Iterative, Evolutive , Parallel	Y (analysis and evaluation)	Y (analysis and evaluation)	P-MCES	Biomedical model, Psychological paradigm, Engineering paradigm
NIATx Model	Gustafson *et al.*	2016	Gustafson DH Jr, Maus A, Judkins J, Dinauer S, Isham A, Johnson R, Landucci G, Atwood AK. Using the NIATx Model to Implement User-Centered Design of Technology for Older Adults. JMIR Hum Factors 2016;3(1):e2	USA	Center for Health Enhancement Systems Studies, Department of Industrial and Systems Engineering, University of Wisconsin - Madison	Agency for Healthcare Research and Quality, Epic Systems Corporation	Health (Behavior change, ehealth, older adults)	Designer, developers	Iterative, Evolutive , Parallel	Y (analysis and evaluation)	Y (analysis and evaluation)	P-M	Psychological paradigm, Engineering paradigm
Integrate, Design, Assess, and Share (IDEAS) Framework	Mummah *et al.*	2016	Mummah SA, Robinson TN, King AC, Gardner CD, Sutton S. IDEAS (Integrate, Design, Assess, and Share): A Framework and Toolkit of Strategies for the Development of More Effective	USA	Stanford Prevention Research Center, Department of Medicine, Stanford University School of Medicine and	NIH, Nutrition Science Initiative and Stanford Child Health Research Institute	Health (Behavior change, ehealth)	Investigators and industry partners	Iterative	Y (analysis and evaluation)	Y (analysis and evaluation)	P-MCES	Biomedical model, Psychological paradigm, Engineering paradigm

			Digital Interventions to Change Health Behavior. J Med Internet Res. 16 déc 2016;18(12):e317.		Behavioural Science Group, Institute of Public Health, University of Cambridge								
Chronic Disease mHealth App Intervention Design Framework	Wilhide III et *al.*	2016	Wilhide III CC, Peeples MM, Anthony Kouyaté RC. Evidence-Based mHealth Chronic Disease Mobile App Intervention Design: Development of a Framework. JMIR Research Protocols. 16 févr 2016;5(1):e25.	USA	WellDoc Inc	None	Health (Behavior change, mHealth apps, chronic diseases)	Researchers, practitioners, health care policy	Iterative, Evolutive	Y (analysis and evaluation)	Y (analysis and evaluation)	P-M	Psychological paradigm, Engineering paradigm
Three-Phase Human-Centered Design Methodology	Harte *et al.*	2017	Harte R, Glynn L, Rodriguez-Molinero A, Baker PM, Scharf T, Quinlan LR, et al. A Human-Centered Design Methodology to Enhance the Usability, Human Factors, and User Experience of Connected Health Systems: A Three-Phase Methodology. JMIR Hum Factors. 2017;4(1):e8.	Ireland	National University of Ireland Galway	EU’s Seventh Framework Programme for Research	Non-Health (engineering)	Designers, researchers, policy makers, users	Iterative Evolutive	Y (analysis and evaluation)	Y (analysis and evaluation)	P-S	Engineering paradigm
DREAM-GLOBAL Framework	Maar *et al.*	2017	Maar MA, Yeates K, Perkins N, Boesch L, Hua-Stewart D, Liu P, et al. A Framework for the Study of Complex mHealth Interventions in Diverse Cultural Settings. JMIR Mhealth Uhealth. 2017;5(4):e47.	Canada	Laurentian University	The Canadian Institutes of Health Research, Grand Challenges Canada, and the Global Alliance for Chronic Diseases	Health (mobile health interventions)	Researchers, designers, policy makers, stakeholders	Iterative	Y (analysis and evaluation)	Y (analysis and evaluation)	P-MCE	Biomedical model, Psychological paradigm, Engineering paradigm

Processes and recommendations for creating mHealth apps for low-income populations	Stephan *et al.*	2017	Stephan LS, Dytz Almeida E, Guimaraes RB, Ley AG, Mathias RG, Assis MV, et al. Processes and Recommendations for Creating mHealth Apps for Low-Income Populations. JMIR Mhealth Uhealth. 2017;5(4):e41.	Brazil	Fundação Universitária de Cardiologia, Porto Alegre	None	Health (mhealth apps for low-income populations)	Designers, developers, researchers	Iterative, Evolutive	Y (analysis and evaluation)	Y (analysis and evaluation)	P-MCES	Psychological paradigm, Engineering paradigm
ACTS model	Mohr *et al.*	2017	Mohr DC, Lyon AR, Lattie EG, Reddy M, Schueller SM. Accelerating Digital Mental Health Research From Early Design and Creation to Successful Implementation and Sustainment. J Med Internet Res. 2017;19(5):e153.	USA	Northwestern University, Chicago	US National Institute of Mental Health	Health (mental health, ehealth)	Researchers, stakeholders	Iterative, Evolutive, Parallel	Y (analysis and evaluation)	Y (analysis and evaluation)	P-MCES	Psychological paradigm, Engineering paradigm
User-centered design process	Vilardaga *et al.*	2018	Vilardaga R, Rizo J, Zeng E, Kientz JA, Ries R, Otis C, et al. User-Centered Design of Learn to Quit, a Smoking Cessation Smartphone App for People With Serious Mental Illness. JMIR Serious Games. 2018;6(1):e2.	USA	Duke University, Durham	National Institute of Drug Abuse	Health (smoking cessation, mental health)	Researchers,developers, stakeholders	Iterative	Y (analysis and evaluation)	Y (analysis and evaluation)	P-MC	Psychological paradigm, Engineering paradigm
8-Step Scoping Framework	Davidson *et al.*	2019	Davidson R, Randhawa G, Cash S. Identification of Complex Health Interventions Suitable for Evaluation: Development and Validation of the 8-	UK	University of Bedfordshire, Luton	University of Bedfordshire, Luton	Health (complex interventions)	Researchers, clinicians, and users	Iterative	Y (analysis and evaluation)	Y (analysis and evaluation)	P-MCE	Biomedical model, Psychological paradigm, Engineering paradigm

			Step Scoping Framework. JMIR Res Protoc. 2019;8(3):e10075.										
TUDER	Wang *et al.*	2019	Wang Y, Fadhil A, Lange JP, Reiterer H. Integrating Taxonomies Into Theory-Based Digital Health Interventions for Behavior Change: A Holistic Framework. JMIR Res Protoc 2019;8(1):e8055	Germany	University of Konstanz, Konstanz	SMARTAC T	Health (digital health interventions)	Researchers, clinicians, government stakeholders	Iterative, Evolutive	Y (analysis and evaluatio n)	Y (analysis and evaluatio n)	P-MCES	Biomedical model, Psychological paradigm, Engineering paradigm
